# Empfehlungen zur Versorgung von Patient:innen mit FOP

**DOI:** 10.1007/s00132-023-04425-y

**Published:** 2023-08-21

**Authors:** L. Seefried, D. Banholzer, R. Fischer, I. Grafe, I. Hüning, R Morhart, R. Oheim, O. Semler, H. Siggelkow, C. Stockklausner, H. Hoyer-Kuhn

**Affiliations:** 1 Osteologie/Klinische Studieneinheit, Universität Würzburg, Brettreichstr. 11, 97074 Würzburg, Deutschland; 2 Sozialpädiatrisches Zentrum, Standort Mitte – Olgahospital, Haus M – Pädiatrie 1, Klinikum Stuttgart, Kriegsbergstr. 60, 70174 Stuttgart, Deutschland; 3 FOP e. V., c/o Ralf Fischer, Frankfurter Landstr. 11a, 61440 Oberursel, Deutschland; 4 Medizinische Klinik und Poliklinik III, Bereich Endokrinologie und Stoffwechsel, Diabetes, Knochenerkrankungen, UniversitätsCentrum für Gesundes Altern, Universitätsklinikum Carl Gustav Carus der Technischen Universität Dresden, Fetscherstr. 74, 01307 Dresden, Deutschland; 5 Zentrum für Metabolisch-Immunologische Erkrankungen und Therapietechnologien Sachsen (MITS), Technische Universität Dresden, Dresden, Deutschland; 6 Institut für Humangenetik, Universitätsklinikum Schleswig-Holstein, Ratzeburger Allee 160, 23538 Lübeck, Deutschland; 7 Triftstr. 12, 82467 Garmisch-Partenkirchen, Deutschland; 8 Institut für Osteologie und Biomechanik, Universitätsklinikum Hamburg-Eppendorf, Martinistr. 52, 20246 Hamburg, Deutschland; 9 Medizinische Fakultät und Uniklinik Köln, Klinik und Poliklinik für Kinder- und Jugendmedizin, Universität zu Köln, Köln, Deutschland; 10 Medizinische Fakultät und Uniklinik Köln, Zentrum für seltene Erkrankungen, Universität zu Köln, Köln, Deutschland; 11 Zentrum für Endokrinologie, Osteologie, Rheumatologie, Nuklearmedizin und Humangenetik, MVZ ENDOKRINOLOGIKUM Göttingen, 37075 Göttingen, Deutschland; 12 Klinik für Gastroenterologie, gastrointestinale Onkologie und Endokrinologie, Universitätsmedizin Göttingen, Göttingen, Deutschland; 13 Abteilung Kinder & Jugendmedizin, Klinikum Garmisch-Partenkirchen, Auenstr. 6, 82467 Garmisch-Partenkirchen, Deutschland

**Keywords:** Fibrodysplasia ossificans progressiva, Heterotope Ossifikationen, Myositis ossificans progressiva, Seltene Erkrankung, Flare-ups, Fibrodysplasia ossificans progressiva, Heterotopic ossifications, Myositis ossificans progressiva, Rare disease, Symptom flare-up

## Abstract

**Hintergrund:**

Bei der Fibrodysplasia ossificans progressiva (FOP) handelt es sich um eine sehr seltene, genetisch bedingte Erkrankung, ausgelöst durch eine „Gain-of-function“-Mutation im *ACVR1*-Gen, welches den Typ-I-Bone-Morphogenetic-Protein(BMP)-Rezeptor ACVR1 („activin A receptor type 1“) – auch bekannt als ALK2 („activin receptor-like kinase 2“) kodiert. Diese Mutation führt zum Auftreten und Fortschreiten heterotoper Ossifikationen (HO) im Weich- und Bindegewebe. Der HO gehen oft Episoden von Weichteilschwellungen, sogenannte Flare-ups voraus. Die für FOP charakteristischen Flare-ups können durch Traumata, Infektionen, Impfungen oder andere medizinische sowie chirurgische Eingriffe induziert werden oder spontan auftreten. Mit fortschreitendem Alter der Patient:innen kommt es bei den Betroffenen aufgrund zunehmender HO zu schwerwiegenden Bewegungseinschränkungen bis hin zur Bewegungsunfähigkeit, die mit einer verkürzten Lebenserwartung einhergeht. Ein erstes charakteristisches klinisches Anzeichen für FOP ist die angeborene Fehlbildung der Großzehen [25] mit valgischer Achsabweichung, die bei fast allen Patient:innen auftritt. Um die Diagnose zu sichern, ist eine molekulargenetische Analyse des *ACVR1*-Gens möglich.

**Ziel der Empfehlungen:**

Ziel der vorliegenden Handlungsempfehlungen ist es, einen Überblick über die notwendigen Voraussetzungen und Bedingungen für die Versorgung von Patient:innen mit FOP zu geben und durch eine bessere Verfügbarkeit von Wissen insgesamt einen positiven Beitrag für Patient:innen mit FOP zu leisten. Um dies zu erreichen, werden relevante Aspekte bei der Versorgung der sehr seltenen Erkrankung FOP vorgestellt, von der initialen Diagnose bis zur Betreuung in der Regelversorgung, basierend auf dem Wissen der Autor:innen (deutsches FOP-Netzwerk) und den internationalen FOP Treatment Guidelines. Die hier vorgestellten Empfehlungen richten sich an alle Akteur:innen und Entscheidungsträger:innen im Gesundheitswesen und sollen darüber hinaus der Information von Betroffenen und der Öffentlichkeit dienen.

## Abstract (en)

**Background:**

Fibrodysplasia ossificans progressiva (FOP) is a very rare, severe genetic disorder triggered by a gain-of-function mutation in the *ACVR1* gene that codes for the type I bone morphogenetic protein (BMP) receptor ACVR1 (activin A receptor—type 1), also known as ALK2 (activin receptor-like kinase-2). It leads to the onset and progression of heterotopic ossification (HO) in soft and connective tissue. HO is often preceded by episodes of soft tissue swelling or flare-ups. Flare-ups, characteristic of FOP, may be induced by trauma, infection, vaccination, or other medications, as well as surgical procedures or may occur spontaneously. As patients age, they develop severe mobility limitations due to progressive HO formation, including immobility, causing a shortened life expectancy. FOP’s first characteristic clinical sign is the congenital malformation of one or both big toes with valgus axis deviation, which is present in almost all patients. To confirm the diagnosis, molecular genetic analysis of the *ACVR1* gene is possible.

**Aim of the recommendations:**

This white paper aims to provide an overview of the necessary prerequisites and conditions for the care of patients with FOP and positively contribute to patients with FOP by improving the overall availability of knowledge. To achieve this, relevant aspects of the care of the very rare disease FOP are presented, from the initial diagnosis to the care in regular care based on the authors’ knowledge (German FOP network) and the international FOP Treatment Guidelines. The recommendations presented here are addressed to all actors and decision-makers in the health care system and are also intended to inform patients and the public.

Die Fibrodysplasia ossificans progressiva (FOP) ist eine schwere, genetisch bedingte Erkrankung, die zu fortschreitender heterotopen Ossifikation (HO) im Weich- und Bindegewebe führt und durch angeborene Fehlbildungen der Großzehen gekennzeichnet ist. Angesichts der Seltenheit der Erkrankung gibt es noch ein erhebliches Verbesserungspotenzial in der Diagnostik und Versorgung der Betroffenen in Deutschland. Als wesentliches Kriterium für eine optimierte Betreuung gilt eine allgemeine Steigerung des Bekanntheitsgrades von FOP und die Vermittlung von Wissen bezüglich Besonderheiten in der (Akut‑)Versorgung.

## Krankheitsbild FOP

**Figure 1:**
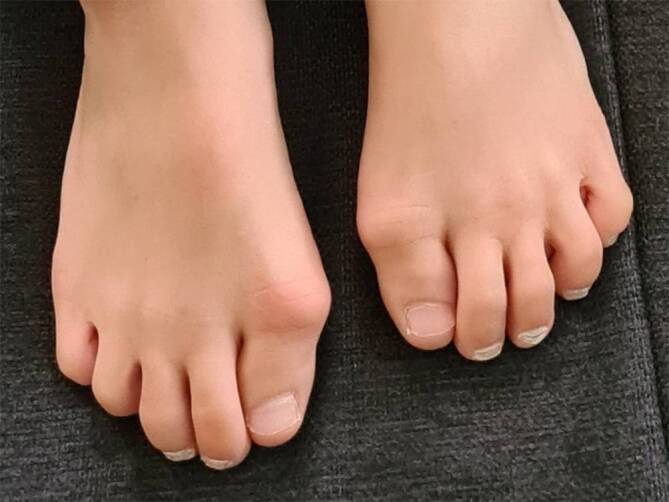

**Figure 2:**
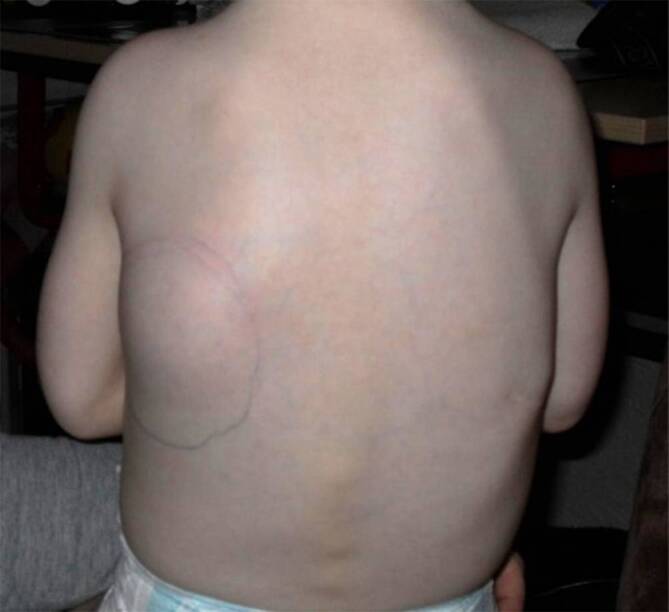

Bei der Fibrodysplasia ossificans progressiva (FOP, Münchmeyer-Syndrom, Myositis ossificans progressiva) handelt es sich um eine sehr seltene genetische Erkrankung, die schätzungsweise 1–1,4 pro 1 Mio. Menschen betrifft [2, 15, 18]. Die FOP ist charakterisiert durch angeborene Skelettfehlbildungen und progressive, kumulative und irreversible heterotope Ossifikationen (HO, Myositis ossificans), ein Prozess der endochondralen Ossifikation, der Weich- und Bindegewebe durch heterotopen Knochen ersetzt [7, 23]. Die glatte Muskulatur und die Herzmuskulatur sind nicht von der HO betroffen [19]. Zu den typischen klinischen Merkmalen zählen die bei fast allen Patient:innen vorhandene mono- oder bilaterale Fehlbildung der Großzehen von Geburt an ([25]; Abb. 1), und fortschreitende, plötzlich auftretende Weichteilschwellungen im Vorfeld der HO ([17]; siehe Abb. 2).

Verursacht wird FOP durch eine „Gain-of-function“-Mutation im *ALK2/ACVR1*-Gen, welches einen BMP-Typ-1-Rezeptor kodiert [24]. FOP wird autosomal-dominant vererbt, entsteht aber meist durch ein spontanes De-novo-Mutationsereignis. Es gibt nur sehr wenige bekannte Fälle mit einer familiären Vorgeschichte [20, 21, 24].

Der irreversiblen HO gehen oft sporadische und unvorhersehbare Episoden von Weichteilschwellungen, Schmerzen, Bewegungseinschränkungen, Steifheit und Wärmegefühl voraus, die als Flare-ups bezeichnet werden [17]. In vereinzelten Fällen treten HO unbemerkt und ohne Flare-ups auf, dennoch beeinträchtigen sie fortlaufend die Mobilität der Patient:innen durch eine Zunahme der Verknöcherung [17]. Die entstehenden Ossifikationen treten meist in einem typischen Muster auf, beginnend im Schulter‑, Hals- sowie Kopfbereich und von dort nach kaudal und distal fortschreitend. Flare-ups und nachfolgende HO, die die Hüfte betreffen, gehen mit der größten Bewegungsbeeinträchtigung einher und dauern in der Regel länger an als andere Flare-ups [17]. Durch das Voranschreiten der Erkrankung entstehen körperliche Einschränkungen und Pflegebedürftigkeit [3]. Patient:innen mit FOP benötigen im Verlauf ihres Lebens zunehmend Unterstützung bei der Durchführung alltäglicher Aktivitäten sowie Hilfsmittel [3]. Die mediane Lebenserwartung betroffener Patient:innen mit FOP liegt bei 56 Jahren (Spanne: 3–77 Jahre); dies ergab eine Analyse umfassender Mortalitätsberichte aus zwei großen FOP-Patient:innenregistern, die den natürlichen Verlauf der Patient:innen mit FOP weltweit umfassen [10].

## Epidemiologie

Die Prävalenz der FOP variiert je nach Land und Region zwischen 0,5 und 1,4 pro eine Million Individuen, was laut Liljesthröm et al. vermutlich im Zusammenhang mit einem unzureichenden Bewusstsein für die FOP und regional unterschiedlicher medizinischer Infrastruktur zusammenhängt [15]. Damit zählt FOP zu den sehr seltenen Krankheiten, den sogenannten „ultra-rare diseases“. Die Zeit vom Auftreten erster Symptome bis zur Diagnosestellung der FOP hat sich von in der Vergangenheit 4 Jahren auf inzwischen im Mittel 1,5 Jahre verkürzt, ist damit aber noch immer unerfreulich lang [22]. Das Krankheitsbild findet aufgrund der Seltenheit oftmals weder in Fachbüchern noch in der Ausbildung der Pädiater:innen, Chirurg:innen, Orthopäd:innen, Onkolog:innen, Rheumatolog:innen und Kinderorthopäd:innen Erwähnung, sodass nach wie vor bei einem recht hohen Anteil der Fälle (ca. 50 %) zunächst andere Diagnosen gestellt werden [22].

## Therapieoptionen

Bislang steht für Patient:innen mit FOP in Europa keine kausale Behandlung zur Verfügung, um Flare-ups zu verhindern oder zu blockieren und schwere Einschränkungen durch die fortschreitende HO zu vermeiden oder zu reduzieren. Das Krankheitsmanagement konzentriert sich auf die Vermeidung von Verletzungen, die symptomatische Behandlung von Flare-ups und eine Optimierung der Restfunktion [19]. Zudem wird dringend geraten, sämtlichen Traumata vorzubeugen und unnötige medizinische/chirurgische Eingriffe sorgfältig zu vermeiden. Flankierende Maßnahmen wie Physiotherapie können, sofern dem Erkrankungsbild angepasst angewendet, sinnvoll sein. Umfangreiche und international anerkannte Empfehlungen für den Umgang mit Patient:innen mit FOP wurden von dem International Clinical Council on FOP (ICC) herausgegeben und entsprechend aktualisiert [9].

## Besonderheiten der Versorgung von Patient:innen mit FOP

### Aktuelle Versorgungsdefizite

Der fortschreitende Verlust von Beweglichkeit, körperlicher Funktionen und folglich Alltagskompetenzen wirkt sich negativ auf die Lebensqualität von Patient:innen mit FOP aus, wie eine internationale Umfrage unter Betroffenen und Angehörigen ergab [1]. Dies rechtfertigt hohe Anstrengungen mit der Zielsetzung einer Vermeidung belastender Krankenhausaufenthalte, Pflegebedürftigkeit und des Versterbens an vermeidbaren Komplikationen. Das Wissen über Besonderheiten der Versorgung von Patient:innen mit FOP muss dringend erweitert werden, um die Voraussetzungen für eine bessere, patient:innennahe Betreuung zu schaffen und unnötige invasive Eingriffe zu vermeiden, die zu einer irreversiblen HO und dem damit verbundenen Mobilitätsverlust führen können. Auch aus Sicht der Betroffenen selbst bzw. ihrer Angehörigen ist die derzeitige Lage suboptimal im Hinblick auf fehlende Anlaufstellen im Akutfall und eine fehlende wohnortnahe Versorgung durch ein informiertes multidisziplinäres Team bzw. Ansprechpartner eines überregional organisierten FOP-Netzwerks mit entsprechender Erfahrung.

Auch wenn die Zeit bis zur korrekten Diagnose in den letzten Jahren deutlich reduziert wurde, suchen Patient:innen bis zur gesicherten Diagnose im Durchschnitt 3,3 Fachärzte auf. Etwas mehr als die Hälfte (52,5 %) der Teilnehmer:innen des internationalen FOP-Registers gaben an, im Verlauf mindestens eine Fehldiagnose erhalten zu haben [14]. Pädiatrie (14 %), Orthopädie (26 %) und Rheumatologie (14 %) sind Fachdisziplinen, die häufig die korrekte Diagnose stellen [5]. Laut der Einschätzung der Autor:innen stellen in Deutschland überwiegend fachärztliche Gruppen aus der Pädiatrie bereits im Kindesalter die korrekte Diagnose (Orthopädie, Onkologie, Rheumatologie).

### FOP-Diagnose und Aufklärung

Die Diagnose erfolgt aufgrund der Seltenheit der Erkrankung oft verzögert [19]. Häufig initial gestellte Verdachtsdiagnosen umfassen unter anderem onkologische Erkrankungen, juvenile Fibromatose, nichthereditäre Myositis ossificans, Klippel-Feil-Syndrom und Hallux valgus [22].

Die frühzeitige Diagnose stellt bei einer Erkrankung wie FOP, die progredient verläuft und zu schwersten körperlichen Einschränkungen sowie zu einer Verkürzung der Lebenszeit führen kann, den wichtigsten Einflussfaktor auf den Verlauf dar. Bereits bei Säuglingen können auffällige Fehlstellungen der Großzehen im Rahmen der Vorsorgeuntersuchungen einen Hinweis auf das Vorliegen von FOP geben. Diese bilateralen Fehlstellungen in Form von verkrümmten oder verkürzten Großzehen sind bereits zur Geburt sichtbar [14]. Die Diagnose der FOP kann klinisch gestellt werden und mittels einer molekulargenetischen Analyse des *ACVR1*-Gens bestätigt werden [11]. Wenn der Verdacht auf FOP besteht, sollten alle elektiven Eingriffe wie Operationen, Biopsien und Impfungen aufgeschoben werden, bis eine endgültige Diagnosestellung erfolgt ist. Wünschenswert wäre, dass Betroffene mit FOP von Haus- bzw. Kinderärzt:innen betreut werden, die bereit sind, FOP-Expert:innen zu konsultieren und ein lokales Behandlungsteam zu koordinieren. Die Patient:innen und ihre Familien sollten zum Zeitpunkt der Diagnose über die deutsche Patient:innenorganisation FOP e. V. informiert werden.

### Versorgung durch ein multidisziplinäres Team

**Figure 3:**
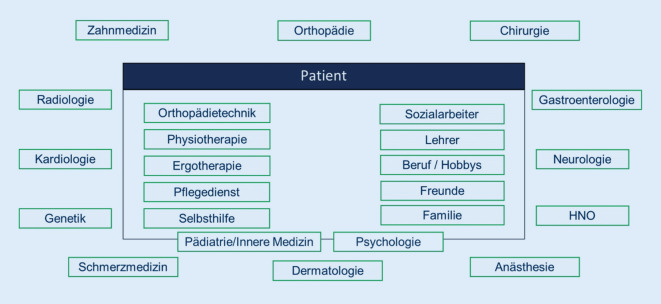

Fehlbehandlungen und invasive Eingriffe können bei Menschen mit FOP zu schweren Folgeschäden führen. Aufgrund der Komplexität der chronisch verlaufenden Erkrankung ist für deren optimale Versorgung gemäß des ICC ein multidisziplinäres Team erforderlich [11]. Hierzu zählen im Umgang mit FOP erfahrene und informierte Expert:innen aus den Bereichen Pädiatrie, Innere Medizin, Kardiologie, Neurologie, Chirurgie, Zahnmedizin, Anästhesie, Hals-Nasen-Ohren-Heilkunde, Physiotherapie, Gynäkologie, Dermatologie und Psychologie (Abb. 3). Die Notwendigkeit einer multimodalen Behandlung auf Basis eines multidisziplinären Teams wird auch durch neue Erkenntnisse unterstützt, die zeigen, das bei Patient:innen mit FOP wichtige kardiopulmonale und neurologische Funktionsstörungen vorliegen können, darunter das Thoraxinsuffizienzsyndrom, pulmonale Hypertonie und neuropathische Schmerzen [12]. Idealerweise sind alle mitbehandelnden ärztlichen Fachgruppen über „do’s & don’ts“ in Zusammenhang mit dem Krankheitsmanagement der FOP, insbesondere im Akutfall, informiert (Tab. 1).

**Table 1:** 

Fachrichtung	Empfehlung
Anästhesie	Erfahrung mit schwierigem Atemwegsmanagement. Im Falle einer geplanten Operation sollte eine fiberoptische Wachintubation verfügbar sein. Bei einem geplanten stationären Aufenthalt ist eine vorherige Kontaktaufnahme zu FOP-Spezialist:innen wünschenswert
HNO	Aufgrund des gehäuften Auftretens von Hörproblemen sollten Kinder regelmäßig daraufhin untersucht werden
Orthopädie/Chirurgie	Frakturversorgung möglichst konservativ mittels Ruhigstellung. Soweit notwendig, Reposition äußerst bedacht und schonend durchführen
	Weiche, bettende Einlagen zur Schmerzreduktion und Stoßdämpfung können hilfreich sein. Individuell und in Absprache mit den Betroffenen und ihren Familien sowie einem Team aus Hilfsmitteltechniker:innen auszuwählen sind weiterhin: Helme für Kinder, Weichschaummatratzen, Pflegebetten, Griffe, Antirutschmatten und Tritthilfen (insbesondere im Bad und Toilettenbereich), Handläufe, Gehhilfen, angepasste Sitzschalen für Rollstühle, höhenverstellbare Tische und Stühle
Physiotherapie	Physiotherapie birgt das Risiko irreversibler Folgen (heterotope Ossifikationen) im Falle einer unsachgemäßen Durchführung [13]. Die Beweglichkeit durch aktive (niemals passive) Übungen zu erhalten, erscheint sinnvoll
Psychotherapie	Kontinuierliche mentale und psychosoziale Unterstützung der Patient:innen und der Angehörigen durch Psychotherapeut:innen ist sinnvoll
Zahnmedizin	Überdehnung des Kiefers sowie intramuskuläre Injektionen von Lokalanästhetika meiden

### Erstvorstellung

Bei einer Erstvorstellung ist die Dokumentation der Vorgeschichte, sowie der bisher erfolgten medizinischen oder chirurgischen Versorgung zu empfehlen. Ebenso aufgenommen werden sollte der bisherige Krankheitsverlauf und das Auftreten von Schüben (Flare-ups). Neben einer allgemeinen körperlichen Untersuchung können eine Erfassung der Vitalparameter und Lungenfunktionsprüfung (ab 6 Jahren) sinnvoll sein. Möglich im Rahmen des Erstgesprächs ist auch die Erfassung von verwendeten Hilfsmitteln, unterstützenden Geräten und Anpassungen.

Eine fotografische Dokumentation z. B. der Kieferöffnung und einer eventuellen Kiefersperre kann bei Bedarf erfolgen. Um den Progress der Erkrankung anhand der körperlichen Funktionsfähigkeit zu erfassen, eignet sich die Anwendung des FOP-Physical Function Questionnaire (FOP-PFQ) Fragebogens als „patient reported outcome measure“ (PROM), bestehend aus 36 Unterfragen, um die Auswirkungen der FOP-Erkrankung auf die körperlichen Funktionen und Aktivitäten des täglichen Lebens zu messen [16]. Eine Empfehlung aus Sicht der Autor:innen ist zudem eine regelmäßige Erfassung des Gelenkstatus. Eine geeignete Möglichkeit stellt dafür der speziell für die Erkrankung entwickelte CAJIS(„cumulative analogue joint involvement scale“)-Erhebungsbogen dar [8].

Um das Fortschreiten der Erkrankung zu erfassen, kann das Volumen des heterotopen Knochens mittels bildgebender Verfahren erfasst werden. Außerhalb von Studien merken die Autor:innen an, dass aufgrund der Strahlenbelastung in der Routineversorgung Bildgebung nur bei akuten Beschwerden, wenn möglich lokalisiert und bei entsprechender klinischer Indikation angewendet werden sollte. Ganzkörperaufnahmen sollten in der klinischen Routine vermieden werden.

### Routineversorgung (Wiedervorstellung)

Die Autor:innen empfehlen jährliche Follow-Up-Termine im Rahmen der Routineversorgung, bei hoher Krankheitsintensität sind gegebenenfalls auch engmaschigere Kontrollen in einem der erfahrenen Zentren des FOP-Netzwerks sinnvoll. Der Krankheitsfortschritt kann dabei mit den zuvor erwähnten Standardparametern und mittels entsprechender Untersuchungsbögen für die Wiedervorstellung erfasst werden.

### Umgang mit Flare-ups

Grundsätzlich ist es wünschenswert, die Therapie von Flare-ups möglichst zeitnah zu initiieren [11]. Angelehnt an die ICC-FOP-Guidelines empfehlen die Autor:innen folgendes Vorgehen für den Fall eines akuten Flare-ups:

- Bei Flare-ups im Bereich des Stamms und Rückens ist eine Behandlung mit nichtsteroidalen Antiphlogistika (NSAID) empfehlenswert, da es zum jetzigen Zeitpunkt für FOP keine zugelassene gezielte Therapiemöglichkeit gibt. Auch eine zusätzliche lokale Anwendung nichtsteroidaler Antiphlogistika ist möglich, um die Flare-ups symptomatisch zu behandeln.
- Bei Flare-ups im Hals‑/Kiefer- und Großgelenkbereich (z. B. Hüfte) sollte möglichst frühzeitig eine Glukokortikoidtherapie (Prednison 2 mg/kg/Tag oral bis zu 100 mg für 4 Tage) erfolgen. Betroffene haben idealerweise eine entsprechende Medikation für 4 Tage zu Hause vorrätig.
- Eventuell ist auch eine i.v. angewendete Glukokortikoid-Stoßtherapie (z. B. Prednisolon 20–30 mg/kg/Tag) über 3 Tage mit stationärer Aufnahme zu erwägen. Auf eine Glukokortikoid-Langzeittherapie sollte verzichtet werden.
- Physiotherapeutische Maßnahmen müssen bei einem Flare-up nicht grundsätzlich eingestellt werden. Vorsichtige, entzündungshemmende Maßnahmen (z. B. Kältetherapie) sind möglich.

### Stationäre Behandlungseinrichtungen und wohnortnahe Versorgung

Aufgrund der Komplexität der Erkrankung haben multidisziplinäre Kompetenzen und der Zugang zu FOP-Expertise einen hohen Stellenwert in der Versorgung der Patient:innen. Die Autor:innen empfehlen allen Behandler:innen Kontakt zum FOP-Netzwerk aufzunehmen bzw. sich diesem anzuschließen.

In der Versorgung von Patient:innen mit seltenen Erkrankungen steht die wohnortnahe Versorgung vor besonderen Herausforderungen. Expert:innen für FOP stehen nicht flächendeckend zur Verfügung. Älteren Patient:innen mit FOP mit einem fortgeschrittenen Krankheitsverlauf fällt es aufgrund ihrer körperlichen Beeinträchtigung zudem schwer, weitere Strecken zum nächstgelegenen Zentrum zurückzulegen. Die wohnortnahe Versorgung sollte daher ausgebaut und die Vernetzung zwischen den Zentren und der wohnortnahen Versorgung gezielt gefördert werden. So könnten ambulante Ärzt:innen in Akutsituationen, z. B. bei einem akuten Flare-up, die Versorgung durch die Gabe von Glukokortikoid sicherstellen. Für den fachlichen Austausch mit FOP-Spezialist:innen in den Zentren sollten die Potenziale der Digitalisierung, z. B. Telekonsile, genutzt und weiter ausgebaut werden.

Für eine qualitätsgesicherte interdisziplinäre und sektorenübergreifende Versorgung fehlt bislang eine regelhafte und gesicherte Finanzierung.

### Rolle und Arbeit der Patient:innenorganisation

Patient:innen mit FOP und Angehörige haben Bedarf an informeller und struktureller Unterstützung, beispielsweise bei der Suche nach medizinischen Ansprechpartner:innen mit Fachexpertise, bei der Beantragung von Hilfsmitteln oder finanziellen Unterstützungsleistungen. In diesem Zusammenhang spielt die Arbeit von Patient:innenorganisationen eine wesentliche Rolle. Bezogen auf Deutschland ist hier der FOP e. V. zu nennen, mit inzwischen 185 Mitgliedern aus 14 Ländern. National gesehen zählt der Verein 140 Mitglieder, davon 45 Patient:innen mit FOP.

Der Verein vermittelt Kontakte zu medizinischen Ansprechpartner:innen mit entsprechender Expertise für FOP, erstellt und liefert Informationen und arbeitet aktiv mit allen fachbezogenen Institutionen zusammen. Ein weiterer Schwerpunkt des Vereins liegt in der Öffentlichkeitsarbeit, der Förderung und inhaltlichen Unterstützung der weltweiten Forschung auf dem Gebiet der seltenen Erkrankung FOP sowie in der Förderung betroffener Mitglieder.

## Krankheitsmodifizierende Therapien bei FOP

Aktuell ist keine krankheitsmodifizierende Arzneimitteltherapie für FOP zugelassen. Schübe lassen sich nur symptomorientiert behandeln. Es befinden sich jedoch einige Wirkstoffe in der klinischen Prüfung [4]. In Kanada wurde zudem im Jahr 2022 das erste krankheitsmodifizierende Arzneimittel zugelassen [6].

Um krankheitsmodifizierende Therapien bei FOP qualitätsgesichert anwenden zu können, sollten in den Zentren grundsätzlich Kenntnisse in der Versorgung von Patient:innen mit seltenen und muskuloskelettalen Erkrankungen vorliegen und Zugang zur FOP-Expertise bestehen. Idealerweise liegen in den Zentren bereits erste Erfahrungen in der symptomorientierten Therapie von FOP sowie mit krankheitsmodifizierenden Therapien in anderen Indikationen vor. Da die Expertise in der Behandlung entscheidend ist, sollten weitere notwendige Behandler:innen nicht ausgeschlossen werden. Eine Beschränkung der Therapieinitiierung und -kontrolle auf die Zentren für Seltene Erkrankungen (ZSE) gemäß § 136c Absatz 5 SGB V wird nicht empfohlen, da nicht alle ZSE einen muskuloskelettalen Schwerpunkt ausweisen und Expertise in der Versorgung von Patient:innen mit FOP auch außerhalb der ZSE besteht.

Bei Initiierung einer krankheitsmodifizierenden Therapie ist insbesondere zu Therapiebeginn eine engmaschige Kontrolle in einem Zentrum zu empfehlen. Die Frequenz der Kontrollen von Patient:innen unter Arzneimitteltherapie sollte sich dabei nach dem individuellen Krankheitsverlauf sowie dem Wirkstoff und seinem Nebenwirkungsprofil richten. Folgeverschreibungen bei gut eingestellten Patient:innen können sowohl durch die Zentren als auch durch wohnortnahe Ärzt:innen auf Grundlage einer Empfehlung des therapieinitiierenden Zentrums erfolgen.

## Fazit für die Praxis

Um die Zeit bis zur Diagnose zu beschleunigen, sollte das Wissen bezüglich FOP(Fibrodysplasia ossificans progressiva)-spezifischer Besonderheiten bei Ärzt:innen erhöht werden.

Die Vernetzung zwischen den Zentren und der wohnortnahen Versorgung ist von Bedeutung. Die Zentren sollten idealerweise in die Versorgung der Patient:innen eingebunden sein.

Folgende Empfehlungen lassen sich ableiten:

- Frühestmögliche Diagnose durch zwei klinische Anzeichen: (1) angeborene Fehlbildungen der Großzehen, (2) akut auftretende Schwellungen, die oft einer HO (heterotopen Ossifikation) vorausgehen. FOP kann durch einen Gentest bestätigt werden.
- Bei Verdacht auf FOP sind invasive Eingriffe zu vermeiden, da diese irreversible HO auslösen können.
- Nach Erstvorstellung empfehlen sich jährliche Verlaufskontrollen.
- Therapien sollten bevorzugt durch FOP-vertraute Ärzt:innen erfolgen und sich nach internationalen Leitlinien richten.
- Zentren sollten idealerweise Erfahrung mit FOP oder seltenen und muskuloskelettalen Erkrankungen haben und im Austausch mit dem FOP-Netzwerk stehen.

## Abkürzungsverzeichnis

ACVR1: Activin A receptor type 1
ALK2: Activin receptor-like kinase 2
BMP: Bone Morphogenetic Protein
CAJIS: Cumulative Analogue Joint Involvement Scale
FOP: Fibrodysplasia Ossificans Progressiva
FOP-PFQ: Fibrodysplasia Ossificans Progressive – Physical Function Questionnaire
HO: Heterotope Ossifikationen
ICC: International Clinical Council
PROM: Patient Reported Outcome Measure
ZSE: Zentren für Seltene Erkrankungen
